# Optimizing photo-mineralization of aqueous methyl orange by nano-ZnO catalyst under simulated natural conditions

**DOI:** 10.1186/s40201-015-0204-0

**Published:** 2015-05-17

**Authors:** Ahed Zyoud, Amani Zu’bi, Muath H. S. Helal, DaeHoon Park, Guy Campet, Hikmat S. Hilal

**Affiliations:** SSERL, Department of Chemistry, An-Najah National University, Nablus, Palestine; College of Pharmacy and Nutrition, University of Saskatchewan, 116 Thorvaldson Building, Saskatoon, S7N 5C9 Canada; Dansuk Industrial Co, LTD. #1239-5, Jeongwang-Dong, Shiheung-Si, Kyonggi-Do 429-913 South Korea; Institut de Chimie de la Matie‘re Condense’ıe de Bordeaux (ICMCB), 87 Avenue du Dr. A Schweitzer, Pessac, 33608 France

**Keywords:** Methyl orange, Contaminant mineralization, Solar simulated light, ZnO nanopowder

## Abstract

**Background:**

Photo-degradation of organic contaminants into non-hazardous mineral compounds is emerging as a strategy to purify water and environment. Tremendous research is being done using direct solar light for these purposes. In this paper we report on optimum conditions for complete mineralization of aqueous methyl orange using lab-prepared ZnO nanopowder catalyst under simulated solar light.

**Results:**

Nano-scale ZnO powder was prepared in the lab by standard methods, and then characterized using electronic absorption spectra, photolumenscence emission (PL) spectra, XRD, and SEM. The powder involved a wurtzite structure with ~19 nm particles living in agglomerates. Photo-degradation progressed faster under neutral or slightly acidic conditions which resemble natural waters. Increasing catalyst concentration increased photodegradation rate to a certain limit. Values of catalyst turn over number and degradation percentage increased under higher light intensity, whereas the quantum yield values decreased. The photocatalytic efficiency of nano-ZnO powders in methyl orange photodegradation in water with solar light has been affected by changing the working conditions. More importantly, the process may be used under natural water conditions with pH normally less than 7, with no need to use high concentrations of catalyst or contaminant. The results also highlight the negative impact of possible high concentrations of CO_2_ on water purification processes. Effects of other added gaseous flows to the reaction mixture are also discussed.

**Conclusion:**

ZnO nano-particles are useful catalyst for complete mineralization of organic contaminants in water. Photo-degradation of organic contaminants with ZnO nano-particles, methyl orange being an example, should be considered for future large scale water purification processes under natural conditions.

## Introduction

Purification of water from hazardous chemicals is an important research area. Organic contaminants, such as industrial dyes, halocarbons and phenol derivatives, are among the main contaminants that demand complete safe removal [1]. Different strategies are being investigated for water remediation, including biological treatment [2, 3], ultra-filtration [4], adsorption methods [5] and others. Such methods may not be favored as they may not cause complete mineralization of the organic contaminant. They simply transfer the pollutant from one phase to another [6]. Advanced Oxidation Processes (AOP) have been proposed as alternative routes for water purification. Among those, oxidation via ozone or hydrogen peroxide has been reported as an effective technique [7–11]. Unfortunately, such methods may be costly, as ozonation demands artificial UV radiations, and hydrogen peroxide is not available free of charge. Contaminant complete mineralization with natural solar light seems to be the most practical process for future water purification. A semiconductor photo-catalyst speeds up the action of light by first absorbing photon and producing electrons and holes [12]. With the abundance of costless solar radiations, a low cost catalyst may thus be useful. Different semiconducting materials, in the powder form, have been assessed as photo-catalysts [13, 14]. TiO_2_ in its anataze form is the most widely used effective photo-catalyst for its high efficiency, photochemical stability, non-toxic nature and low cost. It has been described for degradation of a wide range of organic contaminants [15–22]. Zinc oxide ZnO is a semiconductor with a comparable band gap ~3.2 eV (with wavelength shorter than 400 nm), but has been investigated to a lesser extent in water purification. ZnO is evaluated in many advanced applications such as field-effect transistors, lasers, photodiodes, chemical and biological sensors and solar cells, but to a lesser extent in photo-degradation catalysis [23–26]. One main advantage for ZnO is that it absorbs a larger fraction of solar spectrum, than TiO_2_ does [27]. The performance of ZnO in degrading a number of organic contaminants has been reported [28, 29]. The quantum efficiency of ZnO nano-particles in photo-degrading organic contaminants process is higher than that of TiO_2_ [30, 31], due to its higher absorptivity in waves shorter than 400 nm, which accounts to about 5 % of the reaching solar light.

In this communication the photo-catalytic activity of ZnO powder in complete mineralization of organic contaminants is revisited, focusing on finding optimum conditions that yield highest contaminant removal, for the first time. For application purposes, it is necessary to emulate natural conditions of contaminated waters in terms of pH, low contaminant concentration, low allowable catalyst amount and moderate water temperatures. Influence of CO_2_ and other gas flows on methyl orange photo-degradation process will also be assessed here for the first time. All such reaction parameters will be studied in order to assess feasibility of using ZnO activated photo-degradation of methyl orange in water under simulated natural conditions. To assess feasibility of ZnO catalyst to function on its own in simulated natural waters, multiple use of the added ZnO to photo-degrade fresh methyl orange samples will also be investigated. All such studies are being investigated for the first time.

**Fig. 1:**
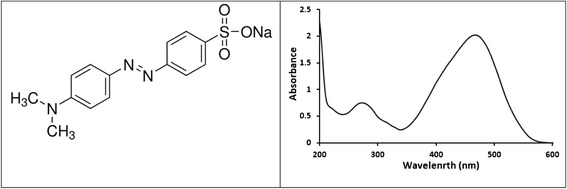
Molecular structure and UV–vis absorption spectrum of methyl orange

Methyl orange, with molecular structure shown in Fig. 1, is a dye that is believed to be mutagenic [32]. It slightly dissolves in water. Its color changes with pH, from yellow (at pH higher than 4.4) to red (at lower pH values), and therefore it is used as an indicator [33]. Methyl orange is also used as a dye in textile industry [34]. It is an example of the widely spread azo dies [35], which are resistant to complete biodegradation [36]. For these reasons, methyl orange is commonly used as a model dye to study in environmental cleanup, and this work is no exception.

In earlier study [37, 38], we reported on using commercial ZnO powders as catalysts in photo-degradation of methyl orange. As mentioned above, this work is intended to find optimum conditions for using ZnO nano-particles in methyl orange photo-degradation under natural water conditions, where ZnO is added to contaminated waters and allowed to function on its own under direct solar light.

## Experimental

### Chemicals

Hydrochloric acid, sodium hydroxide, and methyl orange were purchased from Merck. ZnO powder was prepared in the lab to obtain small particle sizes, as described earlier [39, 40]. A ZnCl_2_ solution (250 mL, 0.25 M) was drop-wise added (within 40 min) to NaOH solution (200 mL, 0.90 M) with continuous stirring. The system was then left to settle, and the supernatant was decanted. The resulting precipitate was washed with water many times to remove any remaining ions. Enough amount of distilled water was added to convert the precipitate into slurry. The slurry was centrifuged at 6000 rpm for 10 min, and the supernatant was carefully decanted leaving the solid catalyst which was dried at 120 °C.

The CO_2_ gas was prepared by adding concentrated HCl solution (5 M) drop-wise to Na_2_CO_3_ solid in a stoppered flask with only one outlet. The outlet was connected with a glass tube bubble the CO_2_ through the reaction mixture at a flow rate 90 mL/L.

### Equipment

A 400 W Osram Tungsten Halogen lamp was used as a source for solar simulator light. The lamp spectrum is a bell curve typical with little (~5 %) in the UV region, just like natural solar light that reaches earth. A light meter (Model lx-102) from Lutron was used to measure the radiation intensity at the reaction mixture surface. A Shimadzu UV-1601 spectrophotometer was used to measure remaining methyl orange concentration using calibration curves, pre-prepared at different pH values, as methyl orange spectra may change with pH value.

The electronic absorption spectra were measured on a Shimadzu UV-1601 spectrophotometer for ZnO powders as suspensions in minimal water amounts. Photoluminescence (PL) Emission Spectra were measured for aqueous suspensions of ZnO powder on a Perkin-Elmer LS50 Luminescence Spectrometer. Excitation wavelength 325 nm was used. XRD patterns were measured on a Philips XRD XPERTPRO diffracto-meter with Cu K_α_ radiation (λ = 1.5418 Å) located in the labs of Dansuk Industrial Co., LTD., South Korea. Field Emission-Scanning Electron Micrographs (FE-SEM) were measured on a Jeol Model JSM-6700 F microscope, in the labs of Dansuk Industrial Co., LTD. South Korea. Atomic absorption spectra (AAS) were used to measure zinc ions resulting from possible degradation of ZnO. The AAS results were measured on an ICE3000 Thermoscientific Atomic Absorption Spectrophotometer equipped with a zinc lamp.

### Catalytic experiment

Photo-catalytic experiments were conducted under direct irradiation from the solar simulator lamp. Water samples pre-contaminated with known concentrations of methyl orange were placed inside a 250 mL beaker. Known nominal amounts of the catalyst ZnO powder were added. The mixture was magnetically stirred with a magnetic bar for 15 min in the dark, to allow adsorption equilibrium and to assess amounts of adsorbed methyl orange on the solid catalyst. The pH of the reaction mixture was controlled by adding drops of dilute HCl or NaOH solutions. The solar simulator lamp, vertically clamped above the solution with an adjustable stand, was then switched on with continuous stirring. The desired irradiation intensity on the mixture surface was achieved by controlling the lamp distance. The reaction time was calculated the time the lamp was switched on. Certain experiments were conducted in duplicate to check the reproducibility of the process.

The reaction progress was followed by measuring the amount of remaining methyl orange with time. This was performed by syringing out small aliquots of reaction mixture at certain times. The aliquots were then centrifuged at high speed (5000 rpm) for 5 min. The liquid phase was then carefully syringed out and analyzed spectrophotometrically at 480 nm.

## Results and discussion

### Zinc oxide powder characterization

**Fig. 2:**
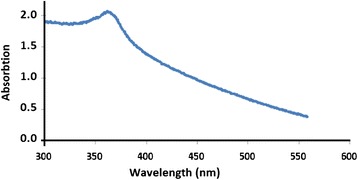
Electronic absorption spectrum for lab-prepared ZnO powder (aqueous suspension)

Solid state electronic absorption spectra were measured for the prepared ZnO nano-powder as aqueous suspension. ZnO showed absorption with a maximum at ~368 nm (equivalent to 3.37 eV), Fig. 2. The wide band gap is presumably due to small average particle size, since smaller particles exhibit wider band gap values [41].

**Fig. 3:**
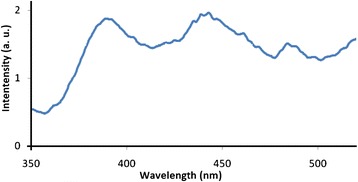
Photoluminescence emission spectrum measured for ZnO powder (aqueous suspension)

The photoluminescence emission spectrum was measured for lab-prepared ZnO powder as dispersion in water, using excitation wavelength 325 nm. The ZnO suspension showed an emission peak at ~385 nm (~3.2 eV), Fig. 3. The other two emission peaks at ~445 nm and ~483 nm are attributed to the presence of oxygen vacancies which cause crystal imperfections, as reported earlier [42]. The value of the band gap is not far different from that measured by electronic absorption spectra discussed above.

**Fig. 4:**
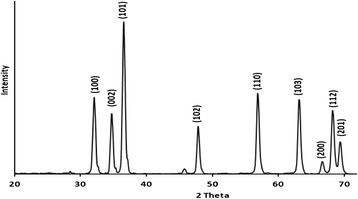
XRD pattern measured for lab-prepared ZnO powder

Figure 4 shows the XRD patterns for ZnO nano-powder. The ZnO clearly involves a wurtzite form, based on comparison with earlier literature results [43]. Based on Scherrer equation approximations, the average particle size was calculated to be ~19 nm.

**Fig. 5:**
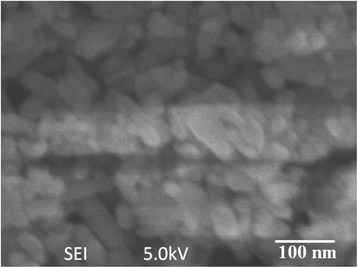
SEM micrograph of lab-prepared ZnO powder

SEM was used to study the surface morphology and to further estimate the particle size of prepared ZnO powders, Fig. 5. The micrograph showed elongated nano-rods (with rice-shape) of ZnO agglomerates. The agglomerates involved nano-particles of less than 20 nm size.

### Methyl orange photo-degradation

Exposure of aqueous solutions of methyl orange to solar simulator lamp, in the presence of ZnO nano-powder, caused appreciable de-colorization of methyl orange solution in soundly short times. Control experiments conducted in the dark, using catalyst while keeping other conditions the same, showed no detectable de-colorization. This means that no degradation occurred in the dark, and that the ZnO powder adsorbs only little fraction of the contaminant. Control experiments conducted with irradiation in the absence of ZnO did not show any noticeable de-colorization with prolonged exposure. This indicates the necessity of the ZnO particles to activate the methyl orange degradation. Using a cut-off filter (that blocks light 400 nm and shorter) caused severe lowering in methyl orange degradation. Collectively, these results indicate that light waves shorter than 400 nm are the driving force for the degradation of methyl orange, and that the ZnO particles are needed to observe the degradation process. Degradation is thus due to the shorter wave length tail available in the lamp light. With a band gap more than 3.2 eV, ZnO powder catalyst employs photons with wavelength shorter than 400 nm in the photo-degradation process.

**Fig. 6:**
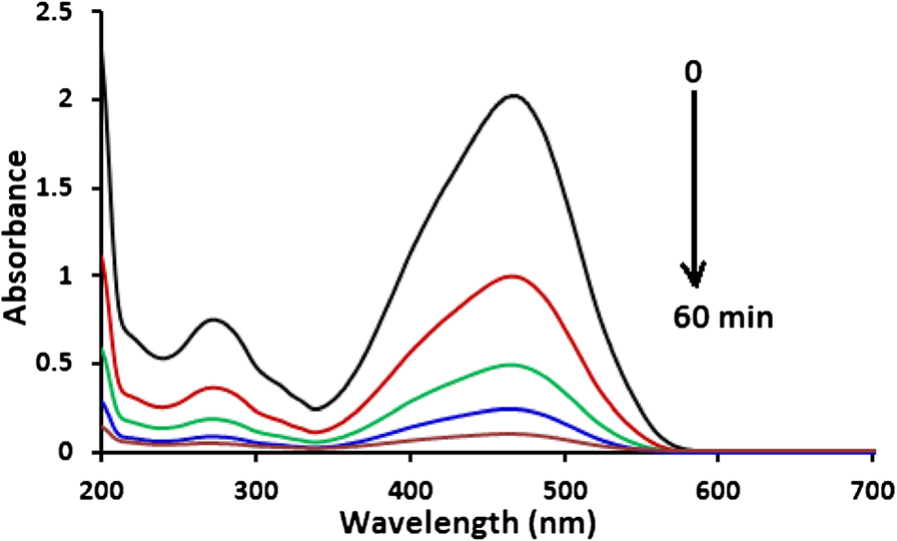
Electronic absorption spectra showing continued mineralization of methyl orange under photo-degradation conditions. Reaction was conducted using methyl orange solution (100 mL, 10 ppm), at 20 °C, under 19.0 mW/cm^2^ total radiation intensity, pH ~7, and ZnO (0.1 g)

Therefore, methyl orange de-colorization here, even in case of incomplete process, is due to complete mineralization of the degraded molecules. This was evident from the disappearance of absorbance bands in the range 200–300 nm (characteristic for the phenyl group) and the range 400–500 nm (characteristic for the azo group). When left for enough time, complete removal and complete mineralization were observed, as shown in Fig. 6 below. Therefore, the reacted phenyl group is believed to be completely degraded leaving no organic products. The reacted azo group is also believed to escape as N_2_ gas [44, 45]. Complete photo-mineralization of methyl orange molecules lost under the working conditions is well documented [37, 46–54].

In order to assess the ability of ZnO to photo-degrade aqueous methyl orange under simulated natural conditions different parameters were investigated here. Effects of different reaction parameters, such as pH, catalyst concentration and contaminant concentration, on rate of photo-degradation were reported earlier [55]. The parameters (initial nominal pH, methyl orange concentration and ZnO amount) have been revisited here together with other new parameters (aqueous CO_2_ concentration, temperature, light intensity and catalyst reuse).

The remaining methyl orange concentrations were plotted with exposure time. The catalytic efficiency is better understood in terms of turnover number, T.N. (degraded molecules/Zn atoms) and quantum yield, Q.Y., (degraded molecules/total incident photons) measured after 30 min exposure to radiation.

#### Effect of pH on photo-degradation process

Because the amphoteric nature of ZnO, it is necessary to study the effect of the pH value on the methyl orange photo-degradation process. This is also necessary as natural waters normally have pH values in the range 5–8 [56, 57].

The pH value affects the ZnO surface OH groups [58], the methylene orange and the aqueous solution species. The pH value affects the generation of the oxidizing species (•OH, O_2_•‾, H_2_O_2_ and HO_2_•) that result in the reaction system [59, 60]. The nature of methyl orange molecule varies with pH value, as stated above. ZnO has a point of zero charge at pH 9.0, above which the ZnO surface is predominantly negatively charged [13]. The electrical properties of the ZnO surface may thus vary with the pH.

**Fig. 7:**
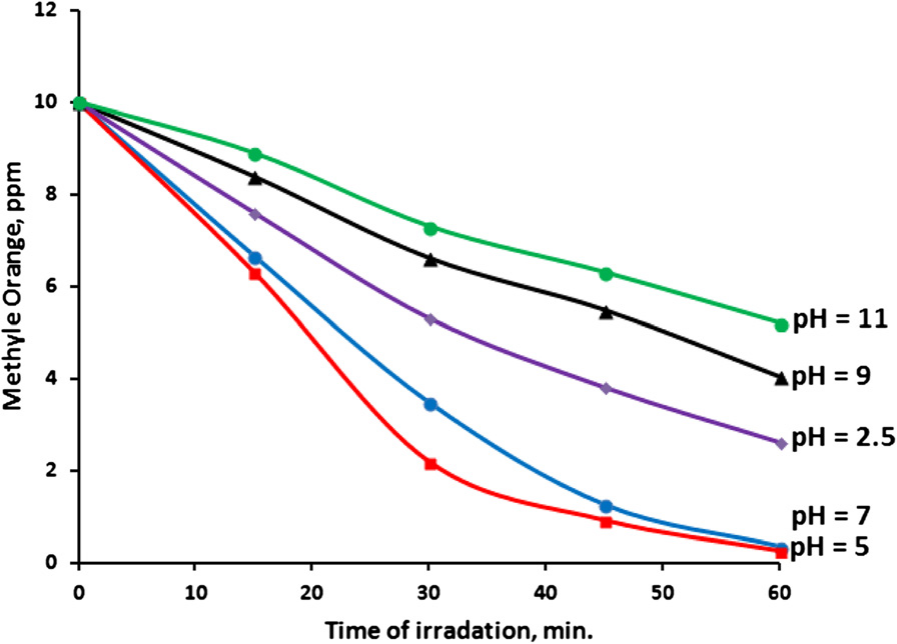
Effect of pH on photo-degradation of aqueous methyl orange. Reactions were conducted using methyl orange solutions (100 mL, 10 ppm) and ZnO (0.1 g) at 20 °C under total radiation intensity 19.0 mW/cm^2^

Experiments were carried out at pH values of (2.5, 5, 7, 9 and 11), Fig. 7. The slightly acidic solution (pH ~5) showed highest T.N. and Q.Y. values, followed by neutral solution. Both solutions with pH 5 and 7 showed complete removal of methyl orange after 60 min of irradiation. As discussed above removal of methyl orange involves complete mineralization, which shows the practicality of using the photo-degradation in natural systems. More acidic or basic solutions (less 2.5 or higher than 9) showed T.N. and Q.Y. values nearly half of those under mild solutions. Table 1 summarizes these results.

**Table 1:** Effect of pH on photo-degradation of aqueous methyl orange. Reactions were conducted using methyl orange solutions (100 mL, 10 ppm) and ZnO (0.1 g) at 20 °C under total radiation intensity 19.0 mW/cm^2^

pH	~7	~5	~2.5	~9	~11
T.N.	1.62 × 10^−3^	1.94 × 10^−3^	1.17 × 10^−3^	8.42 × 10^−4^	6.71 × 10^−4^
Q.Y.	4.36 × 10^−4^	4.28 × 10^−4^	2.92 × 10^−4^	2.12 × 10^−4^	1.74 × 10^−4^
% Degradation	100 %	100 %	73 %	60 %	48 %

In basic media, ZnO becomes Zn(OH)_2_ form with lower semiconducting properties [61]. Under lower pH conditions the lowering in removal efficiency is possibly due to the dissolution of ZnO into Zn^2+^ ions [62], and in highly basic media the ZnO yields zincate ion ZnO_2_^−2^ [63]. The results show that the optimum photo-degradation is close to natural water conditions (neutral to slightly acidic), which adds to the credibility of using ZnO catalyst system. Therefore, unless otherwise stated, all results described here-in-after were obtained under initial nominal pH 7.

#### Effect of temperature

**Fig. 8:**
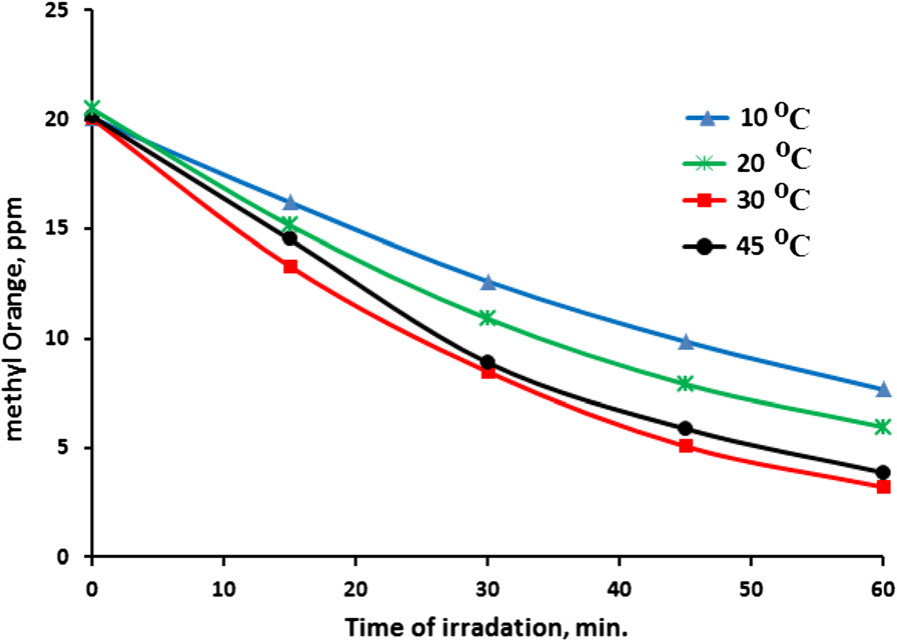
Effect of temperature on methyl orange photo-degradation. Reactions were conducted using methyl orange solution (100 mL, 20 ppm) at different temperatures, using ZnO catalyst (0.1 g) under total irradiation intensity of 19.0 mW/cm^2^ at pH ~7

At temperatures in the range 20–40 °C, the ZnO catalyst showed sound activity, reaching up to 85 % methyl orange removal within 60 min. At lower temperatures, ~ 10 °C, the reaction went slower, reaching up to 65 % removal within 60 min. Figure 8 shows effect of working temperature on the methyl orange phtodegradation reaction profiles. If left for longer time, methyl orange is expected to continue even at lower temperatures. Table 2 summarizes values of TN, QY and degradation% measured at different temperatures. The results show the applicability of using the ZnO system in removing methyl orange with simulated solar light. In this work, unless otherwise stated, all study was performed at 20 °C.

**Table 2:** Effect of temperature on methyl orange photo-degradation. Reactions were conducted using methyl orange solution (100 mL, 20 ppm) at different temperatures, using ZnO catalyst (0.1 g) under total irradiation intensity of 19.0 mW/cm^2^ at pH ~7

Temp.	10 °C	20 °C	30 °C	45 °C
T.N.	1.86 × 10^−3^	2.38 × 10^−3^	2.87 × 10^−3^	2.80 × 10^−3^
Q.Y.	3.52 × 10^−4^	4.28 × 10^−4^	5.44 × 10^−4^	5.29 × 10^−4^
% Degradation	62 %	70 %	84 %	81 %

If higher temperatures (above 45 °C) are used, the reaction goes slower. This is due to possible escape of the oxygen molecules dissolved inside oxygen. The oxygen molecules are involved with the mechanism of photodegradation reaction. Similar results were observed in earlier reports [53].

#### Effect of CO_2_ and other gas flows

**Fig. 9:**
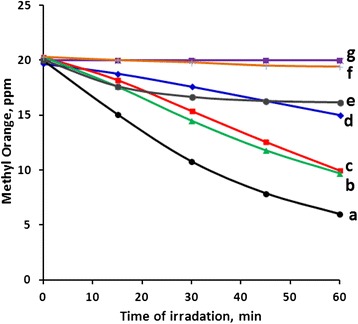
Effect of gas streams on methyl orange photo-degradation. Reactions were conducted using methyl orange solution (100 mL, 20 ppm) under 19.0 mW/cm^2^ irradiation using ZnO (0.1 g) with continuous stirring at 20 °C with different gas flows: (**a**) exposed to air only, (**b**) air flow, (**c**) N_2_ flow open system, (**d**) CO_2_ and air flows together, (**e**) CO_2_ flow, (**f**) closed system with nitrogen flow, (**g**) N_2_ and CO_2_ flows together

The effect of CO_2_ flow on methyl orange photo-degradation was investigated by measuring the reaction profiles for the process while passing a flow of CO_2_ gas. The stream of CO_2_ (90 mL/min), while keeping all other reaction parameters the same, significantly slowed down the removal of the methyl orange, as shown in Fig. 9. Table 3 summarizes values of TN, QY and degradation% while using CO_2_ gas stream. The mode of action of CO_2_ on lowering the reaction rate is not due to lowering the solution pH, as the pH was lowered only slightly, from 6.8 (the nominal used pH) to 6.4, after 60 min. As discussed above the pH in the range 5–8 does not inhibit methyl orange removal process. Alternatively, the mode of action of CO_2_ is thus due to its ability to react with the radicals formed during the photo-degradation process. As reported earlier, *“CO*_*2*_*may interact with some free radical species and may either propagate or inhibit free radical chain reactions”* [64]. In this work the CO_2_ clearly inhibits photo-degradation of methyl orange by interacting with the free radicals believed to be formed during the reaction process. The results reflect a warning signal about the negative impact of possible CO_2_ higher concentrations in natural waters, which would result from increased atmospheric CO_2_ concentration. Having natural waters with higher CO_2_ concentrations may negatively affect future water purification processes.

**Table 3:** Effect of gas streams on methyl orange photo-degradation. Reactions were conducted using methyl orange solution (100 mL, 20 ppm) under 19.0 mW/cm^2^ irradiation using ZnO (0.1 g) with continuous stirring at 20 °C

	Exposed to air only	Air flow	N_2_ flow open system	CO_2_ and air flows together	CO_2_ flow
T.N.	2.87 × 10^−3^	1.72 × 10^−3^	1.44 × 10^−3^	0.75 × 10^−3^	1.03 × 10^−3^
Q.Y.	5.44 × 10^−4^	3.25 × 10^−4^	2.72 × 10^−4^	1.42 × 10^−4^	1.95 × 10^−4^
% Degradation	84 %	52 %	50 %	25 %	19 %

CO_2_ gas stream may arguably affect photodegradation rate by removing oxygen dissolved in the reaction mixture. This was investigated using two continuous streams of CO_2_ and air together. Figure 9 shows that using the air (1000 mL/min) stream with the CO_2_ stream (90 mL/min) did not show significant enhancement in photo-degradation reaction rate. This result further confirms the discussion above, where CO_2_ captures the free radicals necessary for the photodegradation to occur.

Adding a stream of air alone did not increase the reaction rate. Figure 9 shows that the air stream lowered the reaction rate by about 25 % compared to experiments conducted under normal air. In a well known mechanism [40], oxygen molecule is assumed to abstract one electron from the excited ZnO particle leading to O_2_•‾ radical anion species as explained in the equation [e‾ + O_2_ → O_2_•‾]. The O_2_•‾ species is believed to react with the other H_2_O_2_ resulting species to yield the chemically active OH^.^ radical as in the equation [H_**2**_O_**2**_ + O_2_•‾ → OH^•^ + OH^−^ + O_**2**_]. This radical is assumed to react with an organic contaminant molecule and degrade it.

Therefore, the presence of O_2_ in water is necessary. However, as the results here show, excess of O_2_ in water inhibits the photodegradation reaction. This is due to the adverse effect of excess O_2_ which blocks OH^•^ radical as shown in equation [H_**2**_O_**2**_ + O_2_•‾ → OH^•^ + OH^−^ + O_**2**_].

Adding nitrogen to the reaction mixture together with CO_2_ practically stopped the reaction progress (Fig. 9). The effect is perhaps dual in nature, where the CO_2_ behaves as scavenger, as discussed above, while the nitrogen lowers concentration of oxygen that is necessary for the reaction to proceed. Adding a nitrogen flow (240 mL/min) to the reaction mixture, while exposed to atmospheric air, slowed down the process, but did not stop it completely. Nitrogen lowers concentrations of oxygen in the reaction but traces are left therein which are enough for the reaction to occur. All reactions above were conducted under exposure to atmospheric air. This is evident because complete coverage from air while under nitrogen stream caused complete reaction inhibition.

#### Effect of contaminant concentration

**Fig. 10:**
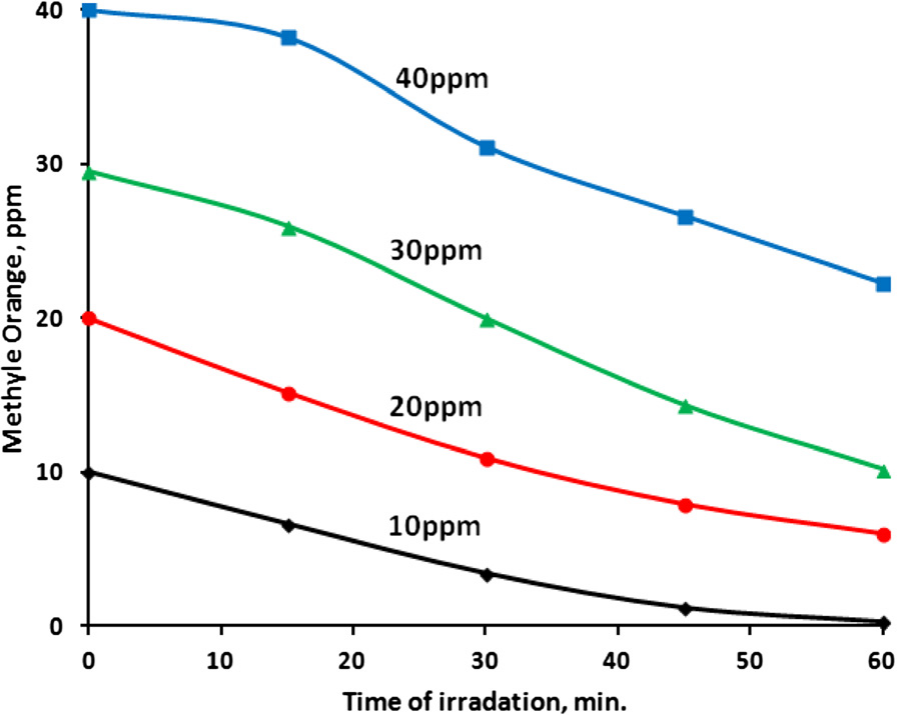
Effect of contaminant concentration on its photo-degradation. Reactions were conducted using different contaminant concentrations in aqueous solution (100 mL), pH ~7, at 20 °C with ZnO (0.1 g) under 19.0 mW/cm^2^ irradiation intensity

The effect of methyl orange concentration (10, 20, 30 and 40 ppm) on its photodegradation process was studied, Fig. 10. Values of percent methyl orange removal, after 60 min, were 100 % for 10 ppm, 70 % for 20 ppm, 66 % for 30 ppm, 45 % for 40 ppm, as summarized in Table 4. Despite the lowering in removal percentage with higher contaminant concentration, the average reaction rate increased. Values of T.N. and Q.Y. show comparable values for the 20, 30, and 40 ppm concentrations. Lower values were observed for the 10 ppm concentration, which is due to the fact that all added methyl orange molecules were mineralized with no more left. This naturally yields lower TN and QY values at lower contaminant concentrations. The results indicate the suitability of ZnO catalyst to function over a relatively wide range of contaminant concentrations. More importantly, the catalyst completely activates mineralization of low contaminant concentrations, which are more likely to occur in nature [37, 53].

**Table 4:** Effect of contaminant concentration on its photo-degradation. Reactions were conducted using different contaminant concentrations in aqueous solution (100 mL), pH ~7, at 20 °C with ZnO (0.1 g) under 19.0 mW/cm^2^ irradiation intensity

	10 ppm	20 ppm	30 ppm	40 ppm
T.N.	1.73 × 10^−3^	2.38 × 10^−3^	2.37 × 10^−3^	2.29 × 10^−3^
Q.Y.	4.36 × 10^−4^	4.28 × 10^−4^	4.48 × 10^−4^	4.17 × 10^−4^
% Degradation	100 %	70 %	66 %	45 %

#### Effect of catalyst nominal amount on photo-degradation process

**Fig. 11:**
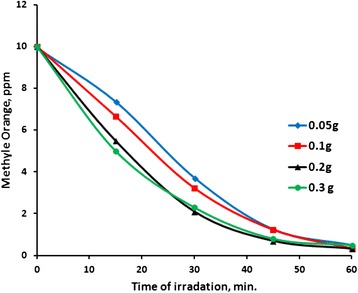
Effect of catalyst amount on methyl orange photo-degradation. Reactions were conducted using methyl orange solution (100 mL, 10 ppm) at 20 °C pH ~7, under 19.0 mW/cm^2^

Catalyst amount may affect photo-degradation processes. The effect of catalyst nominal amount on the photodegradation of methyl orange was investigated using different amounts of ZnO (0.05, 0.10, 0.20 or 0.30 g) in 100 mL solution of 10 ppm methyl orange, under 19.0 mW/cm^2^ irradiation intensity. Figure 11 shows that the average reaction rate was unchanged with catalyst nominal amount. The T.N. value decreased with increasing ZnO amount, as shown in Table 5. This means that the relative efficiency of the catalyst is lowered by increasing catalyst loading. Similar results were observed in other photo-degradation processes [37, 53, 54, 65, 66]. This is attributed to the increased blocking of light with higher catalyst loading. With more catalyst particles, the short wave tail photons are not able to enter the reaction mixture, and the ZnO particles in the reaction mixture do not receive photons. Similar behaviors have been observed in earlier studies [14, 64, 65].

**Table 5:** Effect of catalyst amount on methyl orange photo-degradation. Reactions were conducted using methyl orange solution (100 mL, 10 ppm) at 20 °C pH ~7, under 19.0 mW/cm^2^

	0.05 g	0.10 g	0.20 g	0.3 g
T.N.	3.10 × 10^−3^	1.68 × 10^−3^	9.82 × 10^−4^	9.57 × 10^−4^
Q.Y.	2.93 × 10^−4^	3.19 × 10^−4^	3.72 × 10^−4^	3.62 × 19^−4^
% Degradation	95 %	100 %	100 %	100 %

The QY value showed an increase with increasing nominal catalyst amount at the beginning, but then slightly decreased. Again the lowering in QY is due to the blocking of light by the abundant ZnO particles at the mixture surface which prevent photons from reaching other catalyst sites [14, 37, 53, 54, 65–67]. Agglomeration of smaller ZnO particles into larger ones, in case of higher concentrations, may also play a role, as the total number of surface active sites may decrease [67]. The results indicate that using smaller amounts of catalyst enhanced catalyst efficiency without lowering the average reaction rate or the removal percentage. This is a positive feature of the ZnO catalyst described here, as in case of treating natural waters, smaller amounts of catalyst will be highly favored.

#### Effect of light intensity on the photodegraation process

**Fig. 12:**
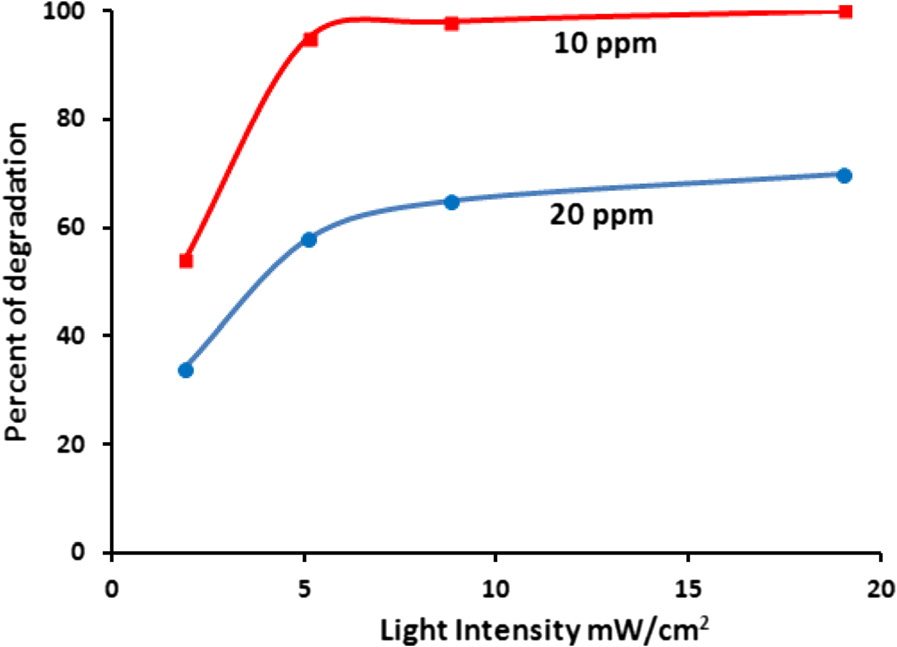
Effect of light intensity on methyl orange photo-degradation. Reactions were conducted using two different methyl orange concentrations in 100 mL solution at pH ~7 with ZnO (0.1 g) at 20 °C

The effect of incident light intensity on photodegradation rate of methyl orange was investigated, using solutions (100 mL each) with two different concentrations (10 and 20 ppm). Experiments were conducted under different light intensities using 0.10 g of ZnO lab prepared powder. In each contaminant concentration, four different light intensities (1.90, 5.12, 8.78 and 19.0 mW/cm^2^) were used. This range includes the average reported daylight intensity of 120 W/m^2^ (12 mW/cm^2^) [68]. Figure 12 shows how methyl orange removal percentage changed with illumination intensity in a period of 30 min for each contaminant concentration. For the 10 ppm concentration case, up to 100 % removal was observed for the higher radiation intensities (5.12, 8.78, and 19 mW/cm^2^), and up to 50 % removal for the lower intensity (1.9 mW/cm^2^), in 30 min. For the 20 ppm concentration case, up to 70 % contaminant removal was observed in 30 min (at the higher radiation intensities, and up to 35 % at the lower irradiation intensity). The results indicate the applicability of using ZnO in removing methyl orange from contaminated water even under radiation intensities lower than normal day light.

Values of TN and QY calculated after 30 min for each case are shown in Table 6. In case of 10 ppm, the lower irradiation intensity (1.9 mW/cm^2^) showed lower TN value (about 50 %) than its higher intensity counterparts (5.12, 8.78, and 19 mW/cm^2^). From the Table and the Figure, it can be seen that under 5.12 mW/cm^2^ or higher, the removal percentage reaches approximately constant value. With lower intensity, lower TN value should be expected, as less photons are available for the catalyst sites. However, as radiation intensity is increased above 5.12 mW/cm^2^, efficiency of light harvesting becomes less. This is evident from values of QY, which decreased when using higher irradiation intensities.

**Table 6:** Effect of light intensity on methyl orange photo-degradation. Reactions were conducted using two different methyl orange concentrations in 100 mL solution at pH ~7 with ZnO (0.1 g) at 20 °C

10 ppm				
	1.90 mW/cm^2^	5.12 mW/cm^2^	8.78 mW/cm^2^	19.0 mW/cm^2^
T.N.	6.96 × 10^−4^	1.5 × 10^−3^	1.58 × 10^−3^	1.73 × 10^−3^
Q.Y.	1.42 × 10^−3^	1.35 × 10^−3^	6.57 × 10 ^−4^	4.36 × 10^−4^
% Degradation	54 %	95 %	96 %	100 %
20 ppm				
	1.90 mW/cm^2^	5.12 mW/cm^2^	8.78 mW/cm^2^	19.0 mW/cm^2^
T.N.	7.96 × 10^−4^	1.86 × 10^−3^	1.99 × 10^−3^	2.38 × 10^−3^
Q.Y.	1.62 × 10^−3^	1.41 × 10^−3^	8.76 × 10^−4^	4.28 × 10^−4^
% Degradation	34 %	58 %	65 %	70 %

For the 20 ppm methyl orange concentration, similar behavior occurred, but the irradiation intensity 5.12 mW/cm^2^ showed highest QY. Collectively the results suggest that it is not necessary to use high irradiation intensities to remove methyl orange from water. This adds to the applicability of using ZnO in natural water purification processes in different environments with different light intensities.

#### Catalyst re-use experiments

**Fig. 13:**
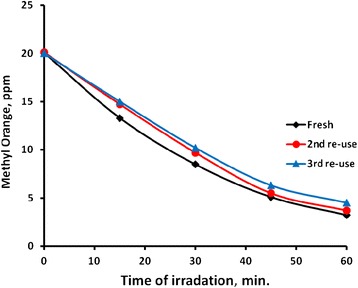
Effect of catalyst reuse on methyl orange photo-degradation. Reactions were conducted using fresh methyl orange solutions (100 mL, 20 ppm) in multiple use of the ZnO catalyst (0.1 g) at 20 °C, pH 7 under 19.0 mW/cm^2^ irradiation intensity

Reusability of the ZnO catalyst for methyl orange photo-degradation in water was studied, by adding fresh amount of methyl orange to the stirred solution after earlier reaction cessation. Figure 13 shows reaction profiles for three time reuse experiments. The Figure shows that at the beginning of each reuse experiment, the measured amount of the methyl orange was 20 ppm. This is due to complete removal of the earlier methyl orange contaminant in the preceding study, as the reaction was left for enough time. In each experiment, after 60 min time of exposure, more than 80 % removal was achieved, showing only low loss of efficiency with reuse.

The amount of Zn^2+^ ions resulting from dissolution of the used ZnO catalyst (with different amounts 0.05–0.3 g per 100 mL solution) during photo-degradation experiments in neutral media was measured by AAS, and was found to be 6 ppm, when the mixture was left overnight. In case of more acidic media the amount was higher, up to 8 ppm. This indicates that only a small fraction of ZnO dissolved under the working conditions. Based on literature [69] the value for solubility of Zn ions resulting from nano-scale ZnO is about 7 ppm. The WHO recommended upper limit for Zn ions is ~5 ppm [70]. The Zn^2+^ ions dissolved in this work is not far from the recommended WHO threshold limits. The results add to the credibility of using ZnO in purification of natural waters.

## Conclusion

Nano-scale ZnO particles can be effectively used as catalysts for complete mineralization of methyl orange in water with solar simulated light. The catalyst can be effectively used under different working conditions (including temperature and pH) that resemble natural waters, and can thus be investigated at larger scale in natural water purification. Adding streams of air, CO_2_ gas, and/or N_2_ gases may affect the reaction progress and may inhibit the reaction.
